# Vascular assessment of liver disease—towards a new frontier in MRI

**DOI:** 10.1259/bjr.20150675

**Published:** 2016-08

**Authors:** Manil D Chouhan, Mark F Lythgoe, Rajeshwar P Mookerjee, Stuart A Taylor

**Affiliations:** ^1^ University College London (UCL) Centre for Medical Imaging, Division of Medicine, UCL, London, UK; ^2^ University College London (UCL) Centre for Advanced Biomedical Imaging, Division of Medicine, UCL, London, UK; ^3^ University College London (UCL) Institute for Liver and Digestive Health, Division of Medicine, UCL, London, UK

## Abstract

Complex haemodynamic phenomena underpin the pathophysiology of chronic liver disease. Non-invasive MRI-based assessment of hepatic vascular parameters therefore has the potential to yield meaningful biomarkers for chronic liver disease. In this review, we provide an overview of vascular sequelae of chronic liver disease amenable to imaging evaluation and describe the current supportive evidence, strengths and the limitations of MRI methodologies, including dynamic contrast-enhanced, dynamic hepatocyte-specific contrast-enhanced, phase-contrast, arterial spin labelling and MR elastography in the assessment of hepatic vascular parameters. We review the broader challenges of quantitative hepatic vascular MRI, including the difficulties of motion artefact, complex post-processing, long acquisition times, validation and limitations of pharmacokinetic models, alongside the potential solutions that will shape the future of MRI and deliver this new frontier to the patient bedside.

Profound hepatic vascular changes occur in chronic liver disease, driving complex phenomena including portal hypertension. Hepatic vascular pathophysiology is complicated by the dual portal venous (PV) and hepatic arterial (HA) blood supply, and the clinical course of chronic liver disease is heterogeneous and often unpredictable. Routine clinical assessment of liver disease assimilates results of serological, non-invasive and invasive tests. The most robust and well-documented biomarker of chronic liver disease prognosis is the hepatic venous pressure gradient (HVPG). The relationship between this invasive surrogate of portal pressure and clinical outcomes underlines the importance of vascular abnormalities in the pathophysiology of liver disease.^1,2^ Non-invasive imaging-based assessment of hepatic vascular parameters would therefore provide clinically meaningful biomarkers for disease staging, therapeutic monitoring and represents a novel opportunity to empower clinical radiologists in defining patient management. In this review, we provide a brief overview of vascular sequelae of chronic liver disease amenable to MRI evaluation and describe existing methods including their applications and challenges in the assessment of hepatic vascular parameters, before concluding with a discussion of future directions.

## LIVER IMAGING—UNMET NEEDS

Liver disease is the fifth most common cause of death in the UK, and there are an estimated 8000 new diagnoses of cirrhosis each year.^3^ Later stage “decompensated” cirrhosis is defined by the presence of ascites, variceal haemorrhage, encephalopathy and/or jaundice, with both ascites and variceal haemorrhage direct sequelae of vascular derangements and portal hypertension.^4^ In compensated patients, the HVPG is the strongest predictor for the development of varices and decompensation, and in patients with a known diagnosis of cirrhosis, each HVPG rise of 1 mmHg leads to a 3% increase in mortality risk.^5^

In spite of this, medical treatments that target hepatic vascular parameters are in their infancy. Licensed treatments include non-cardioselective beta-blockers, effective only in certain patients and often poorly tolerated.^6^ The paucity of robust non-invasive methods for vascular assessment of liver disease has hindered the development and validation of newer, much needed medical treatments.^7^

### Multiple inputs, multiple compartments—the challenge of vascular imaging in the liver

**Figure 1.**
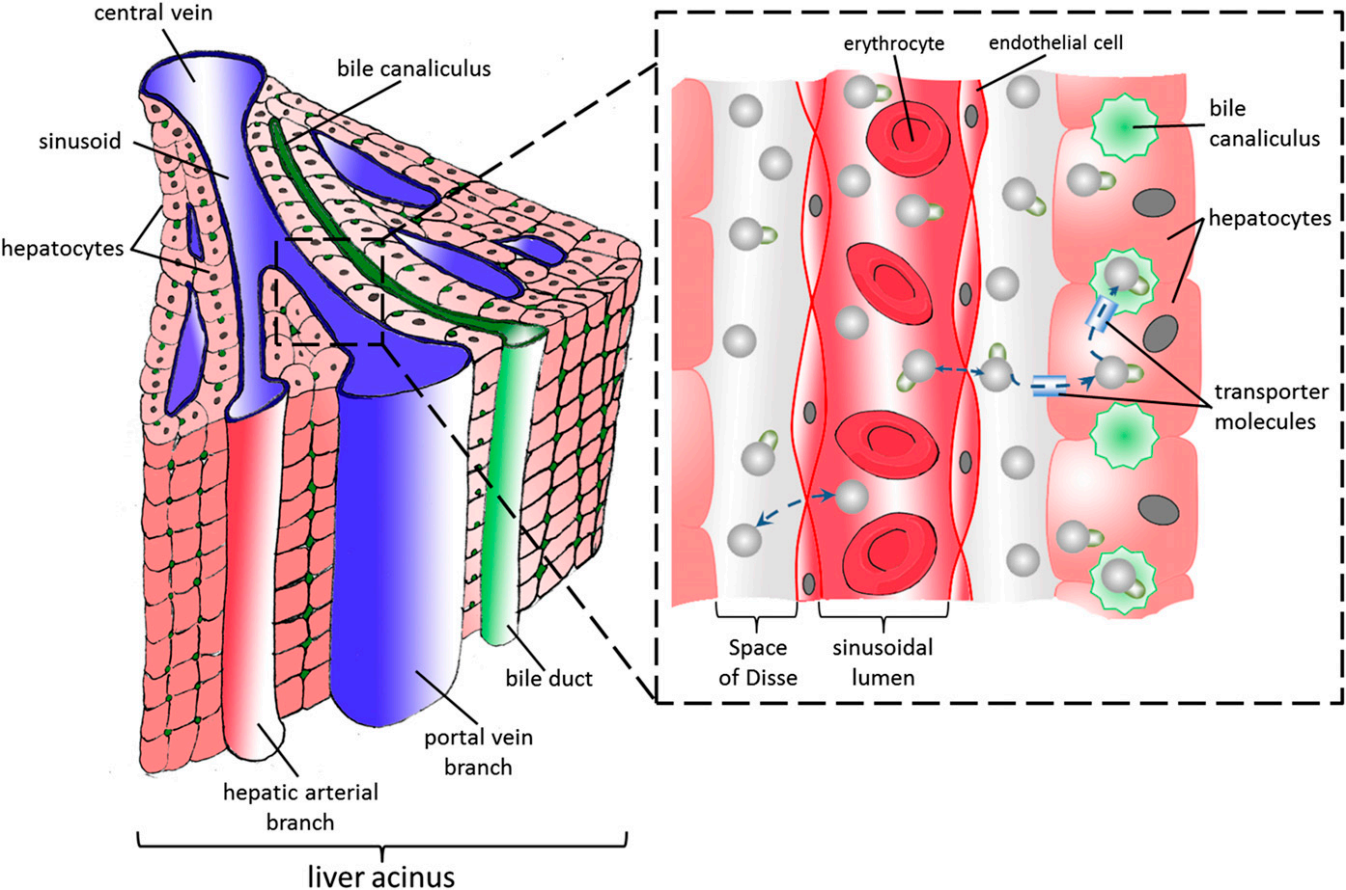
Schematic illustration of the functional organisation of the liver acinus (left). Magnified diagram illustrating the arrangement of sinusoid and space of Disse is shown in the right lower corner. Extracellular contrast agent molecules are demonstrated as spheres on the magnified diagram. Note how these distribute between within the vascular compartment (sinusoidal lumen) and extracellular space (space of Disse). Hepatocyte-specific contrast agents (shown as spheres with an attached flag), distribute between these compartments but are also endocytosed by bile transporters on the surface of hepatocytes before being transported into bile canaliculi.

The healthy liver receives 75–80% of its blood supply from the PV—a low pressure, high capacity vessel—whereas the remaining supply arrives *via* the HA—a vessel of resistance, delivering a smaller volume of blood at higher pressure. Liver parenchyma is organized into “acini” (Figure 1), where afferent PV and HA blood mix in the hepatic sinusoid and drain into an efferent hepatic venule. The sinusoids are flanked almost entirely by hepatocytes but are physically separated from an endothelial cell lining by the “space of Disse”, a separate anatomical compartment into which plasma and low molecular weight compounds (including common extracellular contrast agents) can circulate freely. On the opposing hepatocyte surface but parallel to the sinusoids lie bile canaliculi. These drain bile and products of hepatocyte bile transporters (including hepatocyte-specific contrast agents) proximally into biliary ductules (Figure 1). The healthy liver preserves low pressure within the sinusoids but is tasked with the challenge of being interposed between a mixed high and low pressure input and a low pressure venous output.^8^

### Pressure, flow and resistance—the diseased liver

Fibrosis associated with chronic liver injury is driven by angiogenic factors. Release of contractile factors from vascular smooth muscle cells, sinusoidal endothelial dysfunction and contraction of activated hepatic stellate cells combine to increase intrahepatic parenchymal resistance.^9^ Rises in sinusoidal resistance reduce PV flow and drive formation of extrahepatic collaterals and shunting of splanchnic blood *via* the portosystemic anastomoses. Reductions of PV flow of as much as 60% can be matched by rises in HA blood flow—the so-called “hepatic arterial buffer response”—but this response is impaired in liver disease, so that reductions in PV flow are met with an inadequate response from the HA and a reduction in total liver blood flow.^8^

Deposition of collagen in the space of Disse reduces the volume of the extracellular, extravascular compartment with secondary neovascularization of this fibrotic tissue culminating in reduced effective hepatocyte perfusion and “intrahepatic shunting”. Microvascular thrombi also have the potential to increase the mean transit time (average time for a compound to traverse the parenchyma) for low molecular weight compounds.^10^

### Current gold standards

**Figure 2.**
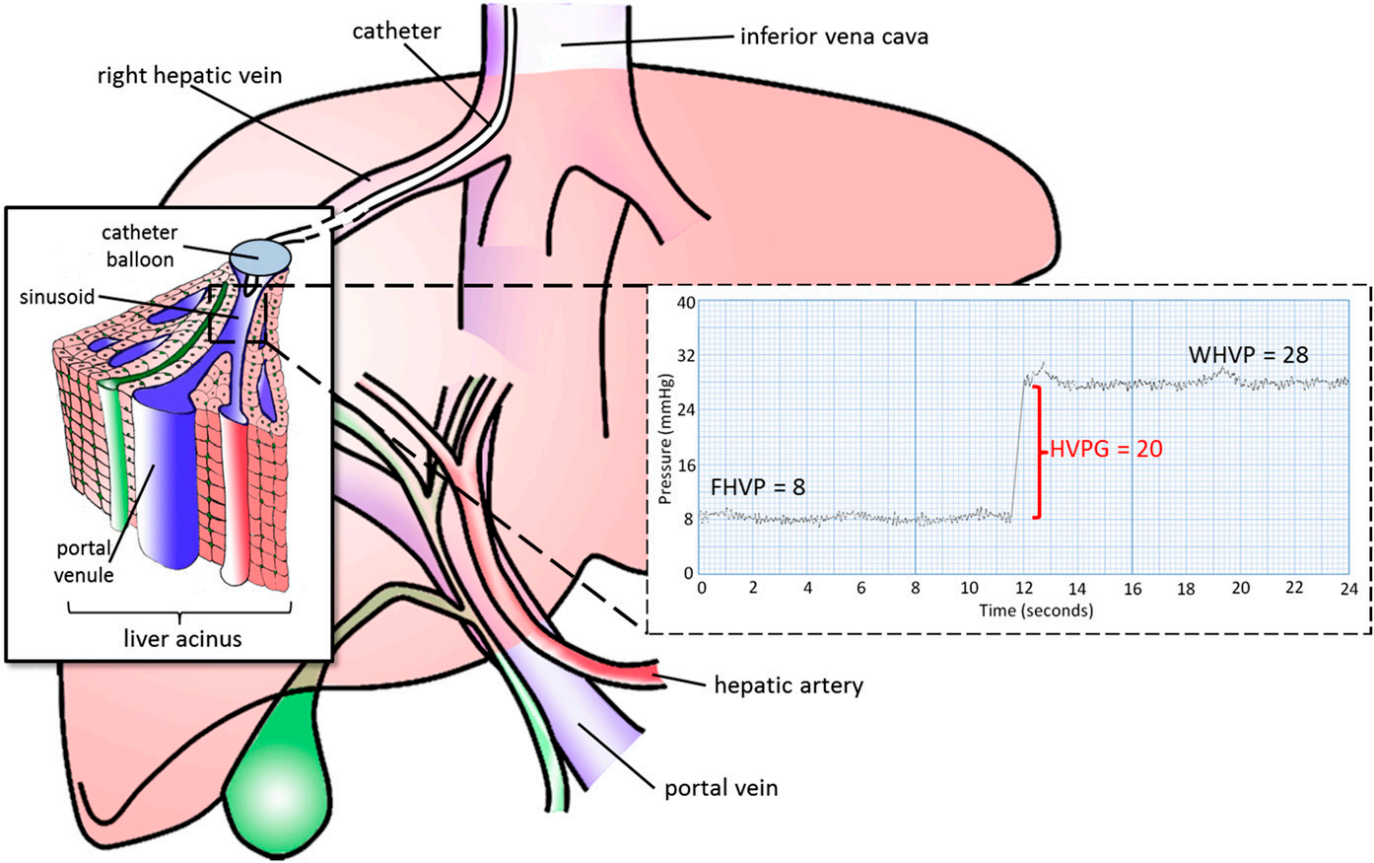
A pressure transducer is advanced *via* the jugular vein, into the right hepatic vein. The pressure trace recorded from the catheter tip known as the “free” hepatic venous pressure (FHVP) is subtracted from the pressure recorded when the balloon is inflated (wedge hepatic venous pressure; WHVP). The latter equates with sinusoidal pressure (recognized to be slightly lower than but directly related to portal venous pressure). Although both of these measurements are subject to variations in intra-abdominal pressure from respiration, the difference of the two—the hepatic venous pressure gradient (HVPG)—eliminates this source of error.

Many well recognized vascular phenomena are visualized on cross-sectional imaging (including postosystemic collaterals, cavernous transformation, varices, mesenteric venous congestion and ascites for example), but these phenomena are qualitative.^11^ Quantitative vascular assessment of liver disease is possible using HVPG but requires invasive fluoroscopic guidance of a pressure transducer into a hepatic vein (Figure 2). Although measurements correlate well with portal pressure, they require calibrated equipment and technical expertise with reported intraindividual variability of as much as 8%, even in specialist centres.^12^

Indocyanine green (ICG) clearance is a widely used reference standard for liver blood flow but requires invasive transjugular hepatic venous sampling and simultaneous peripheral arterial sampling in patients receiving a continuous peripheral ICG infusion. The Fick principle can then be used to estimate effective liver blood flow.^13^ Exclusive hepatic extraction and photometric properties of ICG have yielded less invasive ICG plasma disappearance rate and ICG 15-min retention rate (ICG-R15), which measure hepatic parenchymal function rather than liver blood flow and are potentially subject to error.^14,15^

## APPROACHES TO MR HAEMODYNAMIC IMAGING IN THE LIVER

### Dynamic contrast-enhanced MRI

**Figure 3.**
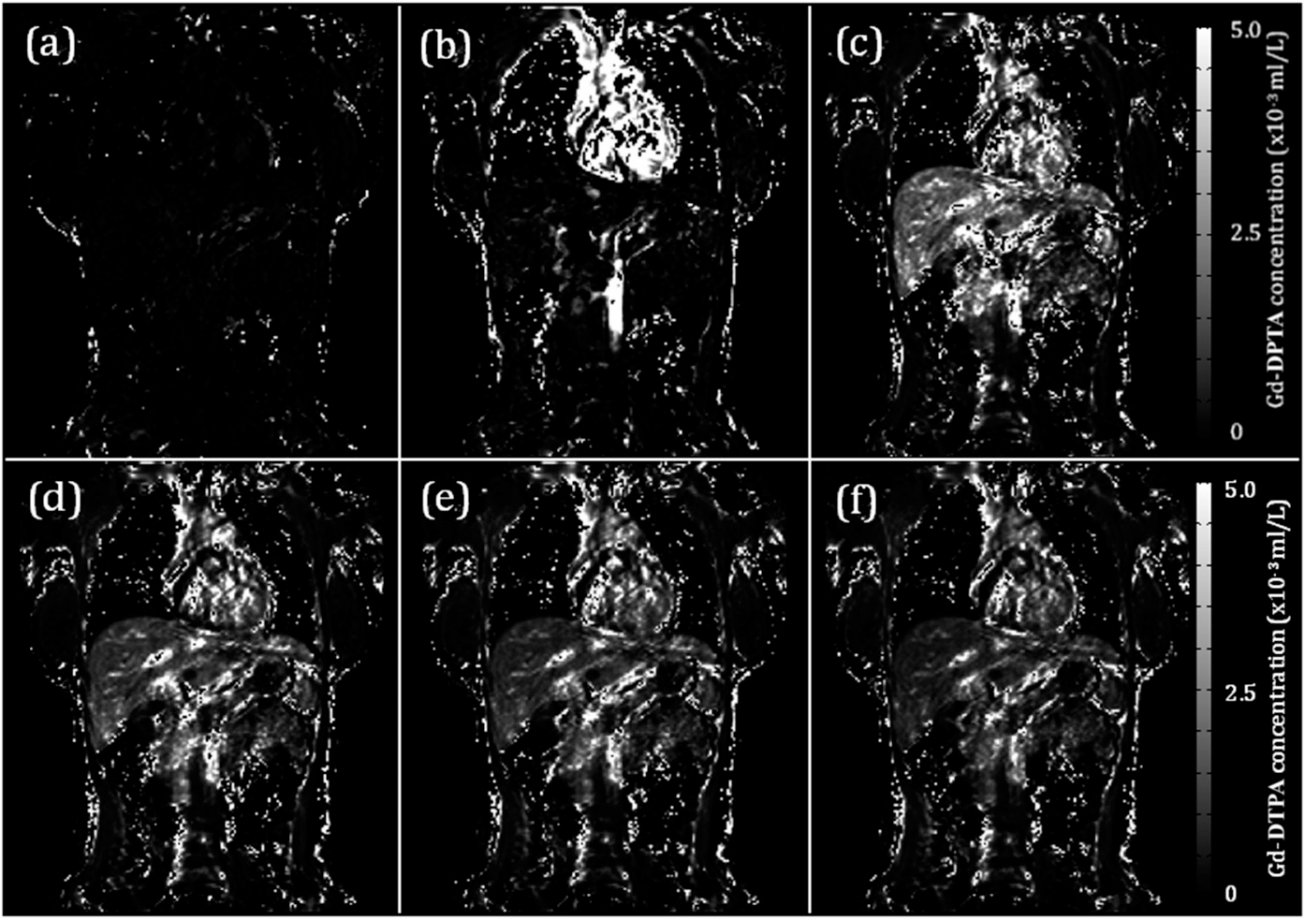
Selected gadolinium chelated with diethylene-triamine-penta-acetic acid (Gd-DTPA) concentration maps from a dynamic contrast-enhanced MRI study. Contrast agent concentration maps for a sample dataset are shown at (a) baseline, (b) 23, (c) 70, (d) 120, (e) 225 and (f) 302 s. Signal on these maps is linear to contrast agent concentration based on the scale on the right. Note the predominantly arterial phase enhancement (b), portal venous phase enhancement (c) and progressive parenchymal washout through to (f).

Multiphase post-contrast imaging is well established in routine liver MRI. By recording serial, high temporal resolution measurements of mean signal intensity (SI) of a region of interest (ROI) after the administration of contrast agent, “dynamic contrast-enhanced” (DCE) MRI can go beyond qualitative evaluation of contrast behaviour and quantify liver perfusion (Figure 3). Gadolinium chelated with diethylene-triamine-penta-acetic acid is used as a contrast agent given its *T*_1_ shortening effect, rapid distribution within the extracellular space and exclusive renal clearance. Contrast agent concentration is, however, not linearly related to SI but linearly related to the reciprocal of a given concentration's *T*_1_ relaxation time thereby complicating analysis.^16^ Formal quantitation therefore requires measurement of the intrinsic *T*_1_ of the tissue and blood within the ROIs and knowledge of the contrast agent *T*_1_ relaxivity.^17^

Early single slice studies in pigs assuming SI linearity were validated using invasive thermal diffusion probes (*r* = 0.91, *p* < 0.01) but were less encouraging once translated into patients (*r* = 0.39, *p* = 0.17).^18,19^ Early formal attempts at contrast agent pharmacokinetic modelling were in the context of liver lesion characterization, where hepatocellular carcinoma demonstrated clear differences in timing and shape of contrast enhancement curves.^20^ CT based methods were later developed to measure “hepatic perfusion index” (a measure of HA fraction within a ROI).^21^

**Figure 4.**
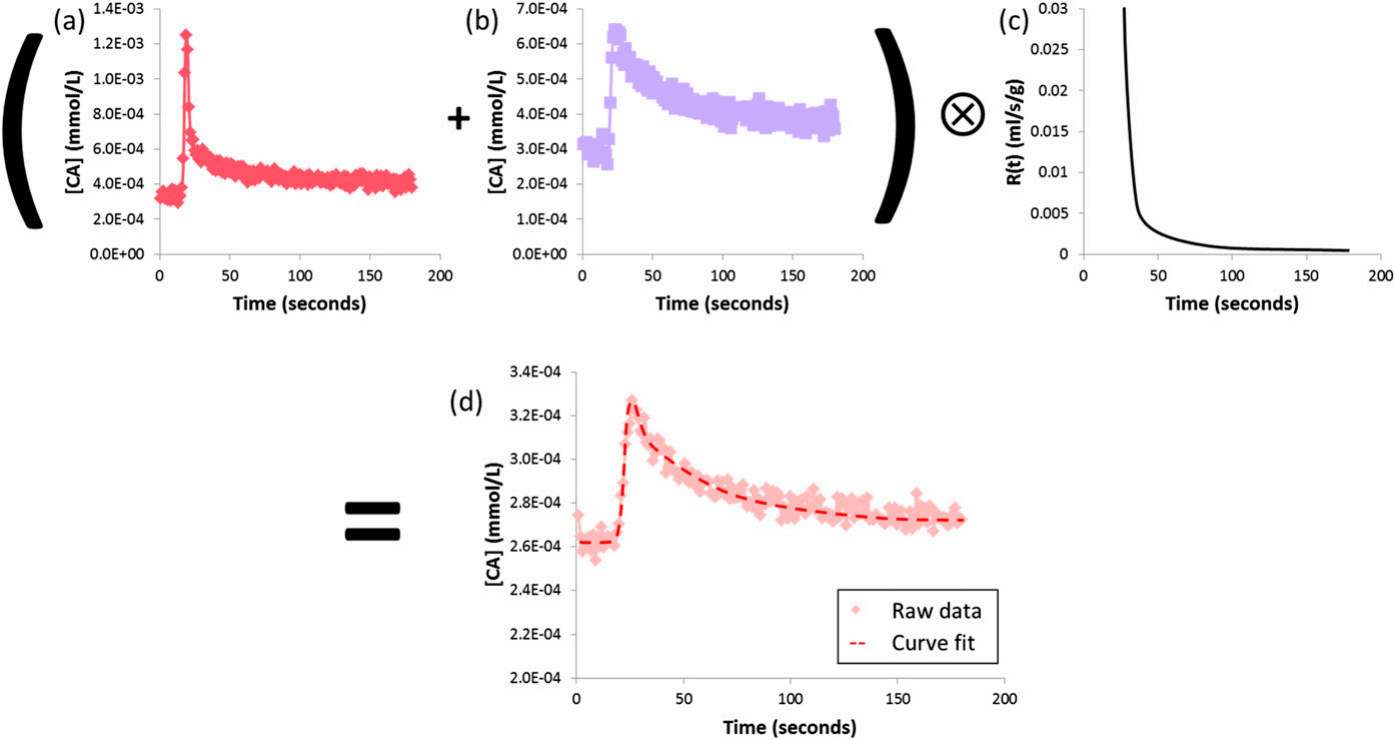
Dual-input single-compartment pharmacokinetic modelling for dynamic contrast-enhanced studies. Regions of interest are used to derive temporal enhancement curves from a single bolus pass of contrast agent. (a) Arterial and (b) portal venous input functions are convolved with (c) an impulse residue function to fit (d) the parenchymal enhancement curve. The transfer constants can be used to estimate absolute arterial and portal venous perfusion (and relative fractions), distribution volume and mean transit time of the contrast agent (CA).

The “dual-input single compartment” model is the most widely adopted model, the dual-input referring the use of aortic and PV enhancement curves (vascular input functions, from ROIs placed over the aorta and PV, Figure 4) and the single compartment referring to the assumption that the parenchymal enhancement arises from a single anatomical space. Invasive microsphere validation in rabbits demonstrated encouraging correlations (PV perfusion *r* = 0.91; HA perfusion *r* = 0.79).^22^ Larger studies in patients with cirrhosis (*n* = 46) have demonstrated significant differences in bulk and relative PV flow between healthy patients and those with cirrhosis. DCE MRI parameters have also been compared with HVPG (correlation with PV fraction *r* = −0.769, *p* < 0.001).^23^

Methodological refinements of the dual-input single compartment model have also been proposed,^23^ including the use of volumetric acquisitions,^24^ correction of arterial input functions,^25^ evaluation of changes in temporal resolution,^26^ alternative approaches to conversion of SI into contrast agent concentration^27^ and the use of alternative breath-holding strategies.^28^

A dual-input dual compartment has also been proposed, with the second compartment reflecting the space of Disse (Figure 1). This has been applied in animal studies using higher molecular weight contrast agents, with encouraging correlations between distribution volume and ICG clearance (*r* = 0.857, *p* = 0.007).^29^ Although the extension of the model in microcirculatory terms is interesting, the dual-input dual-compartment model estimates more parameters from the same data and is therefore more prone to error.

### Dynamic hepatocyte-specific contrast-enhanced MRI

Gadolinium-based contrast agents chelated with hepatocyte-specific receptor ligands [gadobenate dimeglumine (MultiHance^®^; Bracco, Singen, Germany) and gadoxetic acid (Primovist™/Eovist™; Bayer, Berkshire, UK)] produce vascular enhancement followed by progressively increasing hepatocyte *T*_1_ weighted SI as agents are taken up by hepatocyte cell membrane transporters and excreted into the biliary system. Dynamic hepatocyte-specific contrast-enhanced (DHCE) MRI studies thus provide an opportunity to quantify perfusion and hepatocyte function by studying contrast agent uptake.^30^

Greater and more rapid endocytosis of gadoxetic acid has favoured its use in quantitative studies. Studies in rabbits with cirrhosis showed alterations in modelled relative “hepatic extraction fraction” (a quantitative uptake parameter, inferentially related to cell function). Measurements at baseline and post-cirrhosis induction have demonstrated encouraging correlation between the change in hepatic extraction fraction and the change in ICG-R15.^31^ Studies in Child-Pugh class A primary biliary cirrhosis and primary sclerosing cholangitis patients demonstrated significant differences in hepatic extraction fraction and “mean transit time” (but not “input relative blood flow”).^32–34^

Using early post-gadoxetic acid enhancement and dual-input single-compartment modelling, significant differences between HA and PV flow were demonstrated between normal and chronic hepatitic patients.^35^ Furthermore, using raw SI data from the standard five phases of clinical DHCE protocols (baseline, early arterial, arterial, PV and hepatocellular phases), no *T*_1_ measurement and a Patlak model, differences in “uptake rate” and “extracellular volume” across a large cohort of patients with Child-Pugh class A and B and without cirrhosis (*n* = 119) were demonstrated.^36^ The ratio of hepatic parenchymal enhancement during the post-gadoxetic acid hepatobiliary phase to muscle, spleen or baseline SI has also been used to demonstrate good correlations with ICG plasma disappearance rate^37^ and ICG-R15.^38^ Such simplified protocols are undoubtedly attractive for translation into standard clincial practice.

Calculating the ratio of hepatic parenchymal baseline and peak hepatobiliary phase *T*_1_, to generate a “*T*_1_ relaxation time index” or evaluation of raw peak hepatobiliary phase *T*_1_ both represent useful quantitative approaches. These measurements have the potential to define diagnostic thresholds that are transferable between institutions. Such studies have demonstrated changes in *T*_1_-based quantification in the presence of disease and correlations with model for end-stage liver disease (MELD) scores.^39,40^

Both DCE and DHCE-MRI face similar challenges. The non-linear relationship between SI and contrast agent concentration has hampered quantification, complicated post-processing protocols and hindering the generalizability of findings between scanners and institutions. High concentrations of contrast agent can also lead to signal drop out (*T*_2_^*^ dephasing effects),^41^ and poor temporal resolution can introduce significant measurement errors. Standardization of protocols across scanners/institutions can address these problems.

Despite these challenges, MRI offers unparalleled contrast resolution, without compromising patient safety through exposure to ionizing radiation or to large volumes of iodinated contrast media. DCE and DHCE-MRI quantification studies to date have been encouraging, and both approaches have the potential to yield robust quantification.

### Phase-contrast MRI

**Figure 5.**
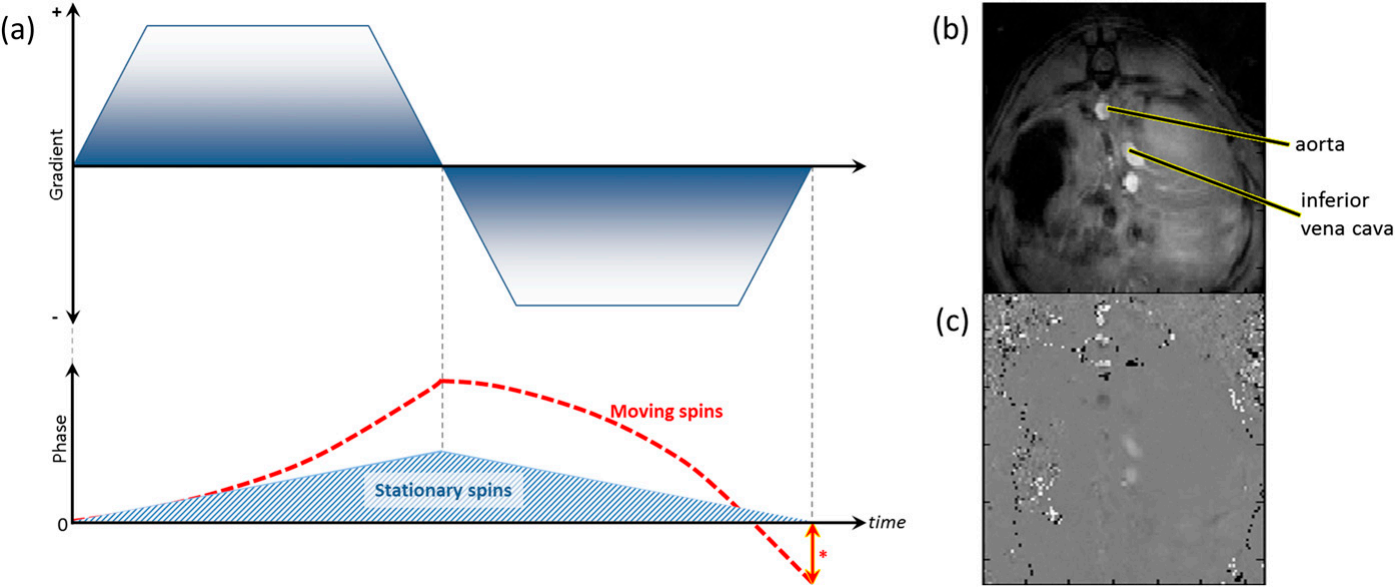
Phase-contrast MRI—(a) schematic diagram of sequence and (b, c) example of phase contrast acquisition images. (a) Bipolar gradients (upper chart) induce phase shifts in both stationary and moving spins (lower chart), but ultimately cancel out the phase shift seen in stationary tissue. The residual signal (*) is proportional to the velocity of moving spins. (b) Anatomical axial “magnitude” image through liver with (c) corresponding phase contrast map. Note the opposing flow directionality (black *vs* white, c) in the descending aorta and inferior vena cava (labelled).

Two-dimensional phase-contrast MRI (PCMRI) sequences are readily available on most clinical MRI systems with established routine clinical applications in cardiovascular and brain MRI for measurement of bulk vessel flow. Unlike Doppler ultrasound, where only a unitary estimation of flow velocity can be made, PCMRI offers high spatial-resolution, operator-independent velocity maps across the vessel lumen. It is based on the principle that all spins in a magnetic field gradient experience shifts in their rotation phase. Moving spins, however, experience phase shifts that are proportional to their velocity. By applying opposing gradients, the stationary tissue phase shifts can be eliminated and a velocity vector map of moving spins (flowing blood) passing through the imaged slice can be created. Summing the vectors over the cross-sectional area of a vessel can estimate of bulk vessel flow (Figure 5).^42^

The first studies investigating hepatic blood flow using PCMRI were undertaken in the PV, with validation studies using a flow phantom^43–45^ or often less successfully with transcutaneous Doppler ultrasound.^46–48^ Comparative reproducibility studies between PCMRI and Doppler ultrasound have demonstrated better PCMRI 1-year reproducibility and reduced variability (combined total liver blood flow coefficient of variation 18% for PCMRI *vs* 33% for Doppler ultrasound).^48^ Multiple PV PCMRI studies have demonstrated reduced PV flow in chronic liver disease and portal hypertension.^47,49–52^ PV PCMRI has been used to demonstrate expected increases in PV flow post-prandially and following transjugular intrahepatic portosystemic shunt (TIPSS) procedures.^43,53^

Very few HA PCMRI studies have been reported likely due to the technical challenges involved, with some normal volunteers and reproducibility studies, and one small patient study.^48,54,55^ There have also been some work demonstrating increased azygous venous flow in the setting of chronic liver disease,^44,45,52^ and reduced azygous flow post-TIPSS procedure^43^ and post-variceal banding.^56^

The technical quality of PCMRI studies has improved over time, but consensus on the clinical value of derived flow measurements has yet to emerge. Elevated PV flow, for example, is associated with variceal haemorrhage, but studies correlating PV flow with gastro-oesophageal variceal grade have been disappointing.^44,49,57^

PCMRI is also beset with technical challenges, which compromise the evaluation of smaller vessels such as the HA: anatomical variations, vessel tortuosity and the requirement for the imaging plane to be perpendicular to the direction of flow in the vessel of interest call for expertise during study planning. Partial voluming, spatial misregistration and “phase-wrapping” (when the velocity of the blood in a vessel exceeds the “velocity encoding” setting for sequence, set by the operator) can also introduce errors.^42,44,49,55^

PCMRI validation is also a challenge: many studies use flow phantoms, but these do not replicate the challenges of *in vivo* imaging.^43–45,50,58^ Transabdominal Doppler ultrasound will only measure velocity, enabling crude estimations of plug flow, but is a poor reference standard with high inter-/intraobserver variability and inferior reproducibility.^46–48,58^

Blood flow when studied as a physiological parameter is classically normalized to organ mass, which can be assessed using anatomical imaging (liver volume correlates well with mass on surgical resection (*r* = 0.954, *p* < 0.001).^51^ Normalized PCMRI flow values are likely to yield more meaningful biomarkers for liver disease but are seldom reported in this way.^51^

Finally, bulk flow-derived biomarkers from two-dimensional PCMRI are valuable because they reduce a large volume of data to a single parameter. This is clinically attractive, as faced with large numbers of complex variables, single parameters can simplify stratification of patients and clinical decision-making.

### Four-dimensional phase-contrast MRI

By acquiring PCMRI data in multiple flow-encoding directions, a three-dimensional image of blood flow can be constructed, which over time can then be used to derive spin (blood) motion streamlines through the cardiac cycle in three dimensions. Combining this with computational flow dynamics, this could be used to derive new and previously unmeasured blood flow parameters including pressure gradient and wall shear stress.^59^

Four-dimensional PCMRI for liver blood flow quantitation is feasible in patients with cirrhosis, but validation with two-dimensional PCMRI and Doppler ultrasound has been unimpressive (*r* = 0.46; *r* = 0.35, respectively).^47,60^ Studies in a small cohort of patients with cirrhosis also failed to show correlation with MELD scores^61^ or differences in patients with known portal hypertension.^62^

Large four-dimensional PCMRI data volumes can be acquired in as little as 20 min, but motion corruption, particularly in the upper abdomen, is a challenge.^59^ To accelerate acquisition times, alternative *k*-space sampling methods such as phase-contrast vastly undersampled isotropic projection reconstruction (PC-VIPR) have been proposed, but these have the potential to introduce artefacts, reduce signal-to-noise ratio^63^ and have uncertain effects on absolute flow quantification.^64,65^ Selection of suitable velocity-encoding settings remains troublesome,^66^ and phase wrapping and noise remain a source of error. Complex post-processing is also a barrier to use.^59^ Nonetheless, the potential to derive alternative haemodynamic parameters will yield new insights into blood flow and will no doubt yield exciting future clinical applications.

### Arterial spin-labelling MRI

**Figure 6.**
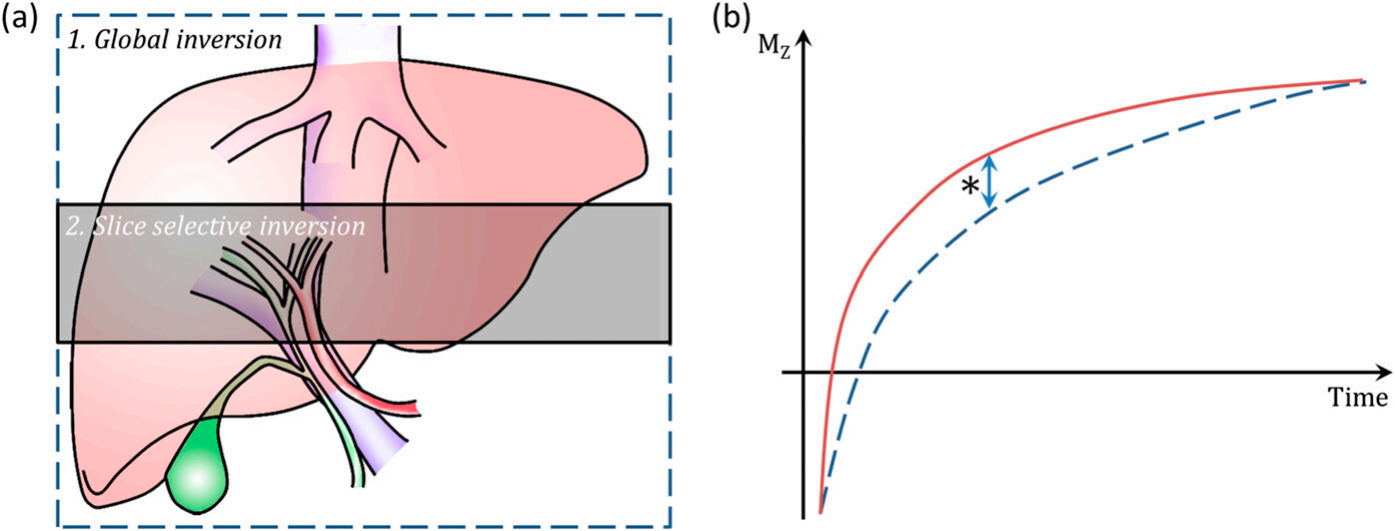
Flow attenuated inversion recovery arterial spin labelling scheme and *T*_1_-based perfusion quantification. Schematic diagram (a), demonstrating arrangement and orientation of consecutive global and slice selective inversions. Note slab sizes are not drawn to scale. The *T*_1_ recovery curves of the slice selective (b, solid curve) and global (b, dashed curve) are shown in (b). The measured difference in *T*_1_ (*, arrow) is dependent on perfusion and can be quantified with knowledge of blood *T*_1_ and the blood–tissue partition coefficient (*λ*).

Arterial spin-labelling (ASL) is based on the generation of a control static signal image and a “labelled”/”flow-sensitised” image, of combined static and magnetized inflowing blood signal. When subtracted, the difference image reflects local perfusion. There are a variety of labelling techniques including pulsed ASL, continuous ASL and pseudocontinuous ASL. The overall ASL signal is dependent on intrinsic tissue and blood *T*_1_, which must be measured for quantification (Figure 6).^67^

Reports of hepatic ASL are sparse, with recently reported pseudocontinuous ASL data in normal volunteers.^68^ Mice liver ASL studies have reported reasonable parenchymal perfusion quantification and changes in perfusion of colorectal liver metastases after administration of vascular disrupting agents.^69^

High-quality validation studies are lacking: studies comparing DCE CT perfusion and ASL have demonstrated positive correlations (*r* = 0.794, *p* < 0.01; *n* = 5),^70^ and clinical studies have been undertaken comparing non-normalized PCMRI PV flow with tissue perfusion.^71^ Child-Pugh class A patients with cirrhosis have also demonstrated reductions in ASL hepatic parenchymal perfusion.^72^

ASL raises specific challenges at labelling, imaging, measuring *T*_1_ and signal modelling stages. Inflowing vessels have variable orientation and consistent labelling strategies, particularly for separation of HA and PV contributions, are essential. Alternative signal modelling approaches may also be required if arterial and PV contributions are to be quantified. Final subtracted ASL signal is small, so that liver motion artefact can easily corrupt the data. Motion correction strategies will be essential for avoiding extended scanning times and the high specific-absorption rate doses required for multiple averages and/or multiple slices.

### MR elastography

Biomechanical imaging methods measure tissue response to an applied physical stress. The tissue response is dependent on the tissue physical properties (viscosity, elasticity and stiffness), and also haemodynamic factors such as tissue perfusion, bulk vessel flow and pressure. Measurement of biomechanical properties thus has the potential to non-invasively probe haemodynamic parameters.

MR elastography (MRE) uses low-frequency mechanical waves propagated through tissues. The waves are imaged using a modified PCMRI sequence with “motion-encoding gradients”. PCMRI data are then used to generate parametric maps of mechanical properties. To produce mechanical stress, a 19-cm plastic disc with a drum membrane is strapped to the patient surface over the right upper abdominal quadrant, under the surface coil. The disc is connected to an active pneumatic driver outside the scanner room. Data acquisition is synchronized with the driver oscillations. Processing occurs at acquisition, with mapping of shear stiffness (pressure applied divided by the ratio of the change in length of the stressed tissue, Young's modulus, kPa) at source. Absolute quantification using ROIs or liver parenchymal segmentation can then be used to record mean stiffness values.^73^ An alternative approach, using different drivers and quantification methods termed “compression-sensitive MRE” has also been proposed.^74^

Pre-clinical studies assessing portal hypertension have been encouraging. A small canine study reported significant correlations between both liver and splenic stiffness and HVPG (*r* = 0.95 and 0.93; *p* < 0.01),^75^ and correlations between swine MRE hepatic and splenic stiffness and colloid infusion volume have been demonstrated (*r* = 0.86 and *r* > 0.90).^76^ Clinical studies have, however, reported modest correlations between hepatic (*r* = 0.44, *p* = 0.017) and splenic (*r* = 0.57, *p* = 0.002) “loss modulus” and pre-transplant HVPG.^77^ Compression-sensitive MRE has been more encouraging, correlating HVPG with hepatic “volumetric strain” (*r* = 0.852, *p* < 0.0001)^78^ and observing changes in splenic viscoelastic constant modulus (G*) (*r* = 0.659, *p* = 0.013),^79^ pre- and post-TIPSS.

The relationship between MRE and the presence and severity of oesophageal varices^77,80^ and hepatic decompensation^81^ has also been positive: larger scale studies will help define formal clinical applications in these contexts.

Finally, liver stiffness changes secondary to haemodynamic changes induced by prandial stress have been compared in patients with chronic liver disease (21.24 ± 14.98%) and normal volunteers (8.08 ± 10.33%).^82^ Correlation between post-prandial PCMRI PV flow and MRE stiffness change (Spearman's *ρ* = 0.48, *p* = 0.013) was unimpressive,^53^ but composite MRE/DCE MRI and MRE/PCMRI parameters have demonstrated improved sensitivity/specificity to the severity of oesophageal varices.^57,80^

Important challenges to assumptions in the biomechanical quantification process lie ahead. The parameters estimated using MRE related to “stiffness” include Young's, loss and shear modulus; divergence and volumetric strain; viscoelastic constants; and decomposed curl to name a few. The nomenclature is confusing and parameters abstract, particularly in the hands of clinicians unfamiliar with biomechanical quantification. The clinical value of this multitude of parameters will become clear from larger scale studies; some parameters of likely of greater value in assessing fibrosis while others more useful in the assessment of haemodynamics.

Ultimately, the biggest challenge to MRE is competition from ultrasound elastography. Two-dimensional parametric mapping is MRE's main advantage, but cost, time and simplicity will always favour sonographic approaches. Future studies that demonstrate superiority of either for clinical applications will clarify the role of each method in the assessment of liver haemodynamics.

## CHALLENGES AND FUTURE DIRECTIONS

The translation of hepatic vascular MRI into the clinical setting faces demanding but not insurmountable challenges, both specific to liver and to quantitative MRI in general. Firstly, the liver experiences complex motion and deformation through respiratory and cardiac cycles: quantitative data must be efficiently registered to derive accurate anatomically localizable data.^83^ Complex post-processing impedes the use of quantitative techniques: new post-processing solutions must be robust, fast and accessible across institutions/scanners; require minimal user input; and be free from costly software/hardware solutions. Acquisition of quantitative data must also be rapid, convenient and cost effective for realistic clinical implementation alongside existing protocols for anatomical imaging.

There is a relative paucity of high-quality validation studies using standards such as HVPG and ICG clearance, although encouraging data are slowly emerging. Through invasive, high-quality validation, the legitimacy of new imaging methods can be better stated and can establish strong foundations for clinical acceptance. Quantification of MR signal is also challenging. DCE, DHCE and ASL methods are reliant on *T*_1_ measurement protocols which themselves are highly variable in the literature. Inaccurate *T*_1_ measurements affect haemodynamic quantification, complicate validation studies and restrict comparisons across institutions/scanners. Conformity of DCE MRI acquisition and analysis protocols has been proposed by international consortia,^84,85^ with liver-specific guidance anticipated in the near future.

Effective use of mathematical models to derive haemodynamic parameters is essential. These must be as simple as possible but yield clinically meaningful haemodynamic parameters. The model-based estimation of parameters must be driven by clinical need rather than mathematical theory. Quantitative data must be useful to radiologists and clinicians: translation into routine imaging protocols will only occur once championed by clinical radiologists and clinicians working in non-academic settings.

Finally, hepatic haemodynamic parameters are inherently complex. Physiological homeostasis of flow, pressure and resistance remains poorly understood. Clinical use of HVPG would favour “pressure” as the key variable for assessment, but this approach is likely too simplistic. Future multimetric imaging protocols must deliver comprehensive haemodynamic assessment through a “one-stop” MRI protocol for perfusion, intrahepatic resistance, hepatic sinusoidal pressure and shunting maps. These could help clinical radiologists identify segmental areas more affected by disease, qualify focal hepatic parenchymal lesions and be used to inform therapeutic interventions ranging from optimizing TIPSS stents to planning and enhancing medical treatments and surgical outcomes.

## CONCLUSION

Chronic liver disease is associated with major vascular changes that to date remain poorly understood but are clear and promising targets for imaging biomarkers. A number of promising quantitative MRI techniques have been developed for hepatic haemodynamic assessment, each with their own strengths and limitations. Future research must include well-designed studies in large cohorts of pre-clinical and clinical subjects to robustly validate new techniques. The future is exciting, as techniques aimed at quantifying fibrosis, biomechanical properties and haemodynamics converge to yield multimetric MRI strategies that equip clinical radiologists with powerful tools to yield useful and meaningful non-invasive biomarkers not only for assessment and prognostication but also in the development of new, evermore personalized treatments for liver disease.

## FUNDING

MDC was funded by the Wellcome Trust (grant WT092186) and supported by researchers at the NIHR University College London Hospitals Biomedical Research Centre. SAT is a National Institute for Health Research (NIHR) senior investigator and MDC is a NIHR clinical lecturer.
